# Comprehensive assessment of quantitative ultrasound and bone mineral density in osteoporosis screening: An empirical study using Mendelian randomization and bibliometrics

**DOI:** 10.1097/MD.0000000000048754

**Published:** 2026-05-15

**Authors:** Zhu Na, Xinyu Guo, Yihua DuMei

**Affiliations:** a Department of Ultrasound, Xianyang Central Hospital, Xianyang, China; b Department of Surgery, The Second Affiliated Hospital of Xi’an Medical University, Xi’an, Shaanxi, China.

**Keywords:** bibliometrics, bone mineral density, Mendelian randomization, osteoporosis, osteoporotic fracture, quantitative ultrasound, validity

## Abstract

Osteoporosis is a systemic skeletal disease characterized by decreased bone mass and microstructural destruction of bone tissue, leading to fragile bones and susceptibility to fractures, significantly affecting the quality of life of patients and increasing the socioeconomic burden. The aim of this study was to investigate the independence of quantitative ultrasound (QUS) of the heel bone in the prediction of osteoporosis and associated fractures, especially its relationship with bone mineral density (BMD). We used Mendelian randomization to analyze the causal relationship between QUS and BMD, osteoporosis and fracture risk by using genetic variants as instrumental variables. The results showed a significant correlation (*P* < .01) between heel QUS metrics and BMD, suggesting that QUS is not independent of BMD in assessing osteoporosis risk. Therefore, the value of QUS needs to be used in conjunction with BMD results in fracture risk assessment. The use of a Mendelian randomization design enhances the inference of causality and avoids bias in traditional observational studies. Therefore, this study employs bibliometric methodology to further supplement existing research on the relationship between QUS measurements of the calcaneus and BMD. In summary, this study emphasizes the importance of integrating BMD in clinical applications and calls for replication of the study in larger samples to further explore the relationship between QUS and BMD in order to improve screening and management strategies for osteoporosis.

## 1. Introduction

Osteoporosis is a systemic skeletal disease characterized by decreased bone mass and destruction of the microstructure of bone tissue, leading to fragile bones and susceptibility to fractures. The disease significantly reduces the quality of life of patients and increases the risk of fracture, which triggers high healthcare costs and socioeconomic burden.^[1]^ Currently, the primary means of screening and diagnosing osteoporosis is dual-energy X-ray absorptiometry (DXA), a technique that assesses an individual’s bone health by accurately measuring bone mineral density (BMD). However, despite its clinical value in detecting BMD, DXA has limitations in comprehensively assessing bone strength and predicting fracture risk. Therefore, there is an urgent need to find more comprehensive and effective assessment tools to enable better understanding and management of bone health and to reduce the potential risks associated with osteoporosis.^[2]^

Among numerous scientific studies, quantitative ultrasound (QUS) of the heel bone is gaining widespread attention and importance as an emerging and promising bone quality assessment tool. Previous research results have shown that QUS is not only effective in assessing and determining bone quality status, but also has a high correlation with osteoporosis and fracture risk.^[3]^ However, it is worth noting that some studies have also raised the thought-provoking idea that the predictive ability of QUS may be independent of the relationship between trabecular bone score and BMD.^[4]^ These controversies prompted us to delve deeper into the relationship between QUS and BMD to provide a basis for further research in this area.

The aim of this study was to analyze the causal relationship between heel bone QUS and BMD, osteoporosis and fracture risk using Mendelian randomization (MR). MR can effectively overcome confounding bias in observational studies by using genetic variants as instrumental variables (IVs), thus providing more reliable causal inferences.^[5]^ The core of the study was to clarify the independence of heel QUS in the assessment of osteoporosis and fracture risk and to explore its relationship with BMD. Through using the method of bibliometrics, the previous studies were summarized, and additional relevant research based on the relationship between QUS of the calcaneus and BMD was supplemented.

The goal of this study was to provide a new reference point for clinical practice and to clarify the potential application of QUS of the heel bone in osteoporosis screening. Through in-depth analysis of the relationship between QUS and BMD, we hope to provide a more scientific basis for early screening and effective management of osteoporosis, thereby improving the prognosis and quality of life of patients.^[2–4]^

## 2. Method

### 2.1. Source of genome-wide association study (GWAS) data

The data used in this study were obtained from the integrative epidemiology unit GWAS database at the University of Bristol (https://gwasmrcieu.ac.uk). For exposure variables, ankle spacing width, ankle spacing width (left), ankle spacing width (right), heel broadband ultrasound attenuation, direct entry, heel broadband ultrasound attenuation (left), heel broadband ultrasound attenuation (right), heel quantitative ultrasound index (QUI), direct entry, heel QUI, direct entry (left), and heel QUI, direct entry (right) are from a study by Ben Elsworth published in a study published in 2018,^[6]^ which included 9,851,867 single nucleotide polymorphisms (SNPs).

For outcome variables, heel BMD data were obtained from a study published by Ben Elsworth in 2018.^[6]^ Osteoporosis with pathological fracture data were obtained from a Finnish database in 2021 femoral neck BMD, forearm BMD, lumbar spine BMD were obtained from a study published by Zheng HF in 2015.^[7]^ Total body BMD, total body BMD (age 0–15), total body BMD (age 15–30), total body BMD (age 30–45), total body BMD (age 45–60), total body BMD (age over 60) data were obtained from a study published by Medina-Gomez C in 2018.^[8]^ Details are shown in Table 1.

**Table 1:** Details of studies and datasets used for analyses.

Exposure /Outcomes	Year	Population	Sample size	nSNPs	GWAS ID
Osteoporosis	2021	European	212,778	16,380,452	finn-b-M13_OSTEOPOROSIS
Osteoporosis with pathological fracture (FG)	2021	European	173,619	16,380,281	finn-b-OSTEOPOROSIS_FRACTURE_FG
Femoral neck BMD	2015	European	32,735	10,586,900	ieu-a-980
Forearm BMD	2015	European	8143	9,955,366	ieu-a-977
Lumbar spine BMD	2015	European	28,498	10,582,867	ieu-a-982
Total body BMD	2018	European	56,284	16,162,733	ebi-a-GCST005348
Total body BMD (age 0–15)	2018	European	11,807	9,351,693	ebi-a-GCST005345
Total body BMD (age 15–30)	2018	European	4180	8,509,502	ebi-a-GCST005344
Total body BMD (age 30–45)	2018	European	10,062	9,656,698	ebi-a-GCST005346
Total body BMD (age 45–60)	2018	European	18,805	10,304,110	ebi-a-GCST005350
Total body BMD (age over 60)	2018	European	22,504	11,932,096	ebi-a-GCST005349
Ankle spacing width	2018	European	265,753	9,851,867	ukb-b-4080
Ankle spacing width (left)	2018	European	146,226	9,851,867	ukb-b-2122
Ankle spacing width (right)	2018	European	146,181	9,851,867	ukb-b-8607
Heel broadband ultrasound attenuation, direct entry	2018	European	265,737	9,851,867	ukb-b-15,851
Heel broadband ultrasound attenuation (left)	2018	European	146,218	9,851,867	ukb-b-5447
Heel broadband ultrasound attenuation (right)	2018	European	146,171	9,851,867	ukb-b-6027
Heel QUI, direct entry	2018	European	265,753	9,851,867	ukb-b-19,234
Heel QUI, direct entry (left)	2018	European	146,226	9,851,867	ukb-b-845
Heel QUI, direct entry (right)	2018	European	146,181	9,851,867	ukb-b-5463
Heel BMD	2018	European	265,627	9,851,867	ukb-b-8875

BMD = bone mineral density, FG = fracture group, GWAS-ID = genome-wide association study identity document, nSNPs = number of single nucleotide polymorphisms, QUI = quantitative ultrasound index.

### 2.2. Instrumental variable selection process

Genome-wide significant SNPs that were independent and highly correlated with the exposure and outcome variables were selected to act as IVs. genome-wide information was derived from the United Kingdom Biobank whole genome sequencing project as a reference.^[9]^ The genome-wide significance threshold for quantitative sonication was set to *P* < 5 × 10^8^, the linkage disequilibrium (*R*^2^) threshold was set to 0.001, and the genetic distance was set to 10 Mb. IVs without any linkage effects were selected from the data. Next, IVs that acted as significant predictors of the outcome variable (*P* < .05) were excluded from the selected IVs. SNPs that were missing from the resultant GWAS dataset were targeted with a cutoff value of *R*^2^ > 0.8 was used to identify proxy SNPs. SNPs were discarded if no suitable proxy SNP was available. The strength of the IVs was verified using the following equation: *R*^2^ × (N − 2)/(1 − *R*^2^), where the *F*-statistic was applied. Here, *R*^2^ represents the proportion of variance in QUS of the calcaneus explained by a given SNP, and N represents the sample size. More precisely, *R*^2^ is calculated using the following formula: *R*^2^ = (2 × *β*^2^ × [1 − EAF] × EAF)/(2 × *β*^2^ × [1 − EAF] × EAF + 2 × SE^2^ × N × [1 − EAF] × EAF). Here, *β* denotes the genetic effect of SNP on QUS of the calcaneus, EAF is the effect allele frequency, SE is the standard error, and N is the sample size; only strong IVs (*F*-statistic > 10) were retained for each exposure of interest. Fourth, we excluded ambiguous versus palindromic SNPs (minor allele frequency > 0.42) whose effects could not be corrected during coordination. MR polytropic residuals and outliers tests were conducted to discard SNPs with potential polytropic effects.

### 2.3. MR

To obtain robust and reliable causal inferences about the effect of QUS on BMD, osteoporosis, and osteoporotic fracture risk factors, we implemented a multiplicative random-effects inverse variance weighted (IVW) analysis in the primary analysis. Sensitivity analyses were conducted using a weighted median (WM) approach with MR-Egger regression, which is not constrained by the zero-intercept and identifies genotype-outcome dose-response relationships that account for pleiotropic effects. However, the MR-Egger method is more sensitive to detecting associations between unobserved genetic variants and confounders in the exposure-outcome association and requires larger sample sizes for the same level of potential exposure variants. The WM approach provides consistent effect estimates when at least 50% of the information in the analysis is derived from valid IVs. Heterogeneity was assessed using Cochran Q test and IVW methods.^[10]^ Heterogeneity was considered absent if the *P* value of Cochran Q > .05. Horizontal pleiotropy was checked using the intercept term obtained from MR-Egger regression. Subsequently, one-by-one culling analysis was performed to assess whether the IVW estimates were biased by a single SNP. The one-by-one culling method was treated as a sensitivity analysis. We looked up each SNP in Phenoscanner (http://www.phenoscanner.medschl.cam.ac.uk/). All statistical analyses were done with the help of R software (version 4.3.2; R Foundation for Statistical Computing, Vienna, Austria), and the TwoSampleMR program package (version 0.5.5; available from the Comprehensive R Archive Network [CRAN]).

### 2.4. Bibliometrics

In this bibliometric analysis, we retrieved 222 publications from the PubMed database, covering various types including original articles, clinical trials, reviews, and others, specifically 116 original articles, 6 clinical trials, 6 reviews, and 114 other types. The initial publication timeframe spanned from 2010 to 2025. After screening for literature from the most recent 15 years, 192 publications were retained. Through the combination of document type filtering and duplicate removal, a final set of 192 publications was included, providing a precise data foundation for subsequent analysis.

## 3. Results

**Figure 1.**
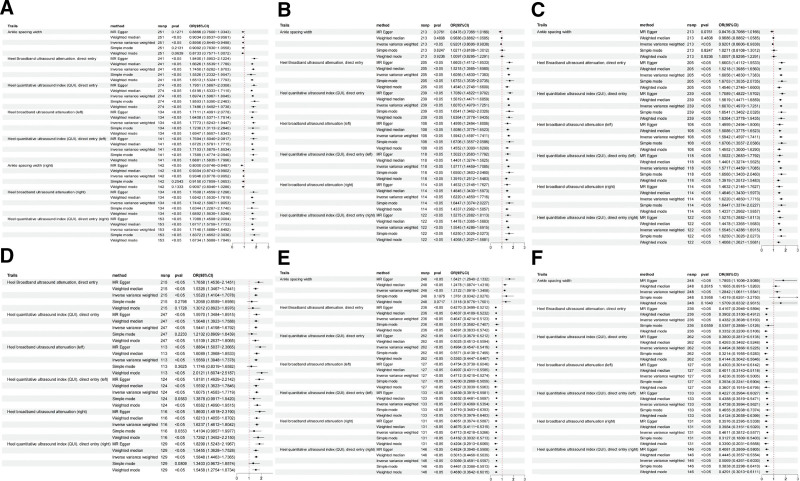
Forest plots analyzing the causal relationships between ultrasound detection indicators and various outcomes using different MR methods. Forest plot of the causal influence of ankle spacing width, ankle spacing width (right), heel broadband ultrasound attenuation, direct entry, heel broadband ultrasound attenuation (left), heel broadband ultrasound attenuation (right), heel QUI, direct entry, heel QUI, direct entry (left), heel QUI, direct entry (right) on (A) total body BMD, (B) lumbar spine BMD, (C) femoral neck BMD, (D) forearm BMD, (E) osteoporosis,(F) osteoporosis with pathological fracture (FG). BMD = bone mineral density, CI = confidence interval, FG = fracture group, IVW = inverse variance weighted method, MR = Mendelian randomization, OR = odds ratio, QUI = quantitative ultrasound index, SNP = single nucleotide polymorphism.

**Figure 2.**
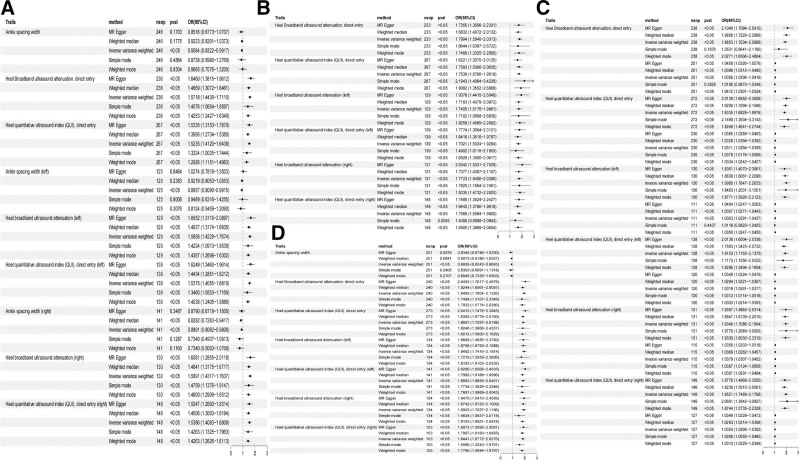
Forest plots analyzing the causal relationships between ultrasound detection indicators and various outcomes using different MR methods. Forest plot of the causal influence of ankle spacing width, ankle spacing width (right), heel broadband ultrasound attenuation, direct entry, heel broadband ultrasound attenuation (left), heel broadband ultrasound attenuation (right), heel QUI, direct entry, heel QUI, direct entry (left), heel QUI, direct entry (right) on (A) total body BMD(0–15), (B) total body BMD(15–30), (C) total body BMD(30–45), (D) total body BMD(45–60). BMD = bone mineral density, CI = confidence interval, IVW = inverse variance weighted method, MR = Mendelian randomization, OR = odds ratio, QUI = quantitative ultrasound index, SNP = single nucleotide polymorphism.

**Figure 3.**
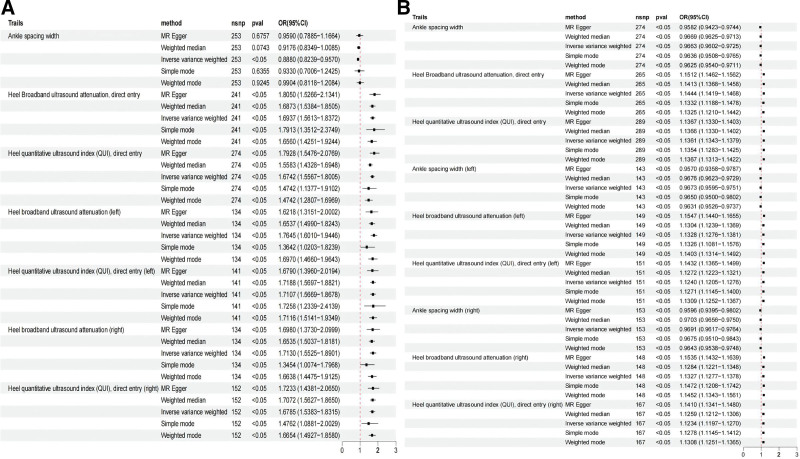
Forest plots analyzing the causal relationships between ultrasound detection indicators and various outcomes using different MR methods. Forest plot of the causal influence of ankle spacing width, ankle spacing width (right), heel broadband ultrasound attenuation, direct entry, heel broadband ultrasound attenuation (left), heel broadband ultrasound attenuation (right), heel QUI, direct entry, heel QUI, direct entry (left), heel QUI, direct entry (right) on (A) heel BMD, (B) total body BMD (over 60). BMD = bone mineral density, CI = confidence interval, IVW = inverse variance weighted method, MR = Mendelian randomization, OR = odds ratio, QUI = quantitative index, SNP = single nucleotide polymorphism.

**Figure 4.**
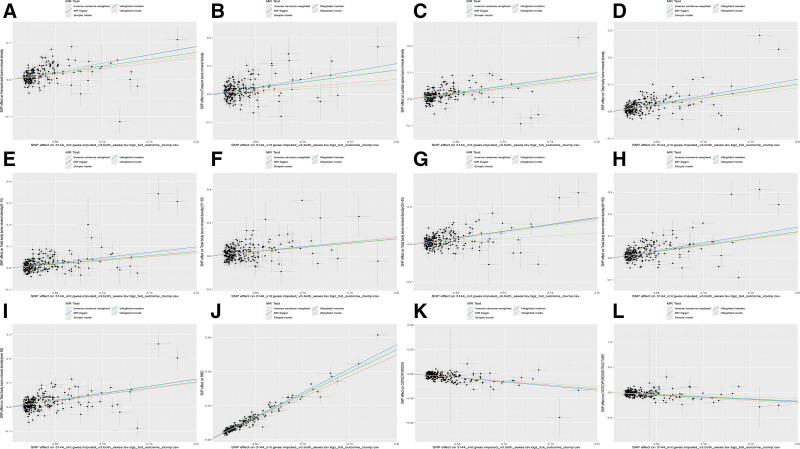
Scatter plots showing the causal relationship between heel broadband ultrasound attenuation, direct entry and the results obtained using different MR methods. Scatter plot of the causal relationship between heel broadband ultrasound attenuation, direct entry on (A) femoral neck BMD(B) forearm BMD, (C) lumbar spine BMD, (D) total body BMD, (E) total body BMD (0–15), (F) total body BMD (15–30), (G) total body BMD (30–45), (H) total body BMD (45–60), (I) total body BMD (over 60), (J) heel BMD, (K) osteoporosis, (L) osteoporosis fracture. The slope of each line corresponds to the causal estimate for each method. The background depicts the relationship between the effect of a single SNP on the outcome (points and vertical lines) and its effect on the exposure (points and horizontal lines). BMD = bone mineral density, MR = Mendelian randomization, SNP = single nucleotide polymorphism.

**Figure 5.**
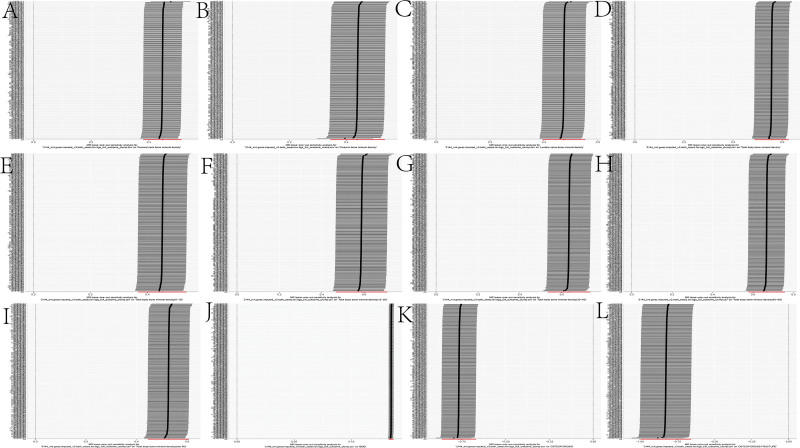
Results of leave-one-out method sensitivity analysis. Leave-one-out sensitivity analysis for the effect of heel broadband ultrasound attenuation, direct entry on (A) femoral neck BMD, (B) forearm BMD, (C) lumbar spine BMD, (D) total body BMD, (E) total body BMD (0–15), (F) total body BMD (15–30), (G) total body BMD (30–45), (H) total body BMD (45–60), (I) total body BMD (over 60), (J) heel BMD, (K) osteoporosis, (L) osteoporosis fracture. BMD = bone mineral density, MR = Mendelian randomization, SNP = single nucleotide polymorphism.

Heel broadband ultrasound attenuation, direct entry included a total of 241 SNPs as IVs, which were used to analyze their association with whole-body BMD (Fig. 1). MR results showed that IVW, MR-Egger, WM, and simple median methods of as an exposure factor were consistent with the direction of the effect (Fig. 4). The results of the IVW method showed a significant association between heel broadband ultrasound attenuation was significantly associated with whole-body BMD (*β* = 0.557, SE = 0.035, odds ratio (OR) = 1.745, 95% credibility interval [CI]: 1.628–1.870, *P* < .001, *F* = 247.833) (Tables 2 and 3). Sensitivity analyses indicated that none of the SNPs, when excluded individually, had a substantial impact on the estimated causal associations (Fig. 5). *P* value for Cochran Q was 1.10e−232 (Table 3). There was no evidence of horizontal pleiotropy (MR_pleiotropy *P* value = .406) (Table 3). The correlations between the remaining QUS indicators and BMD, osteoporosis, and osteoporotic fractures are shown in Figures 1, 2, and 3 and the supplementary materials S1,S2,S3,S4,S5,S6,S7,S8,S9,S10,S11 and S12.

**Table 2:** MR estimates of heel broadband ultrasound attenuation on outcome variable.

Outcome	Method	*β*	SE	OR	or_lci95	or_uci95	*P* value
Femoral neck BMD	MR-Egger	0.453	0.069	1.573	1.374	1.800	< .001
	WM	0.371	0.037	1.450	1.348	1.560	< .001
	IVW	0.447	0.034	1.564	1.464	1.670	< .001
	Simple mode	0.337	0.110	1.400	1.128	1.739	.003
	Weighted mode	0.301	0.051	1.351	1.222	1.494	< .001
Forearm BMD	MR-Egger	0.569	0.099	1.766	1.454	2.145	< .001
	WM	0.427	0.066	1.533	1.347	1.744	< .001
	IVW	0.440	0.049	1.552	1.410	1.708	< .001
	Simple mode	0.188	0.173	1.207	0.859	1.696	.280
	Weighted mode	0.263	0.193	1.301	0.892	1.898	.173
Lumbar spine BMD	MR-Egger	0.507	0.083	1.660	1.411	1.953	< .001
	WM	0.420	0.043	1.522	1.398	1.656	< .001
	IVW	0.474	0.041	1.606	1.483	1.738	< .001
	Simple mode	0.516	0.109	1.675	1.354	2.074	< .001
	Weighted mode	0.375	0.067	1.455	1.275	1.660	< .001
Total body BMD	MR-Egger	0.610	0.073	1.840	1.595	2.122	< .001
	WM	0.508	0.035	1.663	1.554	1.779	< .001
	IVW	0.557	0.035	1.745	1.628	1.870	< .001
	Simple mode	0.440	0.118	1.553	1.233	1.955	< .001
	Weighted mode	0.502	0.038	1.651	1.532	1.779	< .001
Total body BMD (0–15)	MR-Egger	0.498	0.089	1.646	1.381	1.961	< .001
	WM	0.383	0.059	1.467	1.307	1.646	< .001
	IVW	0.452	0.043	1.572	1.444	1.711	< .001
	Simple mode	0.342	0.142	1.408	1.065	1.860	.017
	Weighted mode	0.354	0.070	1.425	1.243	1.635	< .001
Total body BMD (15–30)	MR-Egger	0.551	0.128	1.735	1.351	2.230	< .001
	WM	0.521	0.091	1.683	1.407	2.013	< .001
	IVW	0.587	0.062	1.799	1.594	2.031	< .001
	Simple mode	0.509	0.242	1.664	1.037	2.672	.036
	Weighted mode	0.557	0.138	1.746	1.332	2.288	< .001
Total body BMD (30–45)	MR-Egger	0.744	0.106	2.104	1.708	2.591	< .001
	WM	0.686	0.072	1.986	1.723	2.289	< .001
	IVW	0.634	0.052	1.885	1.703	2.087	< .001
	Simple mode	0.302	0.229	1.353	0.864	2.119	.188
	Weighted mode	0.707	0.103	2.027	1.657	2.480	< .001
Total body BMD (45–60)	MR-Egger	0.717	0.095	2.049	1.702	2.468	< .001
	WM	0.601	0.049	1.824	1.656	2.010	< .001
	IVW	0.667	0.046	1.949	1.780	2.133	< .001
	Simple mode	0.559	0.147	1.749	1.311	2.335	< .001
	Weighted mode	0.584	0.065	1.793	1.577	2.039	< .001
Total body BMD (over 60)	MR-Egger	0.591	0.085	1.805	1.527	2.134	< .001
	WM	0.523	0.047	1.687	1.538	1.851	< .001
	IVW	0.527	0.042	1.694	1.561	1.837	< .001
	Simple mode	0.583	0.144	1.791	1.351	2.375	< .001
	Weighted mode	0.504	0.077	1.656	1.425	1.924	< .001
Heel BMD	MR-Egger	0.141	0.002	1.151	1.146	1.156	< .001
	WM	0.132	0.002	1.141	1.137	1.146	< .001
	IVW	0.135	0.001	1.144	1.142	1.147	< .001
	Simple mode	0.125	0.007	1.133	1.119	1.148	< .001
	Weighted mode	0.124	0.005	1.133	1.121	1.144	< .001
Osteoporosis	MR-Egger	−0.851	0.102	0.427	0.350	0.521	< .001
	WM	−0.769	0.062	0.464	0.411	0.523	< .001
	IVW	−0.766	0.050	0.465	0.421	0.512	< .001
	Simple mode	−0.663	0.185	0.515	0.358	0.741	< .001
	Weighted mode	−0.757	0.103	0.469	0.383	0.574	< .001
Osteoporosis fracture	MR-Egger	−0.875	0.177	0.417	0.295	0.589	< .001
	WM	−0.941	0.117	0.390	0.310	0.491	< .001
	IVW	−0.825	0.087	0.438	0.370	0.519	< .001
	Simple mode	−0.619	0.322	0.539	0.287	1.013	.056
	Weighted mode	−1.093	0.215	0.335	0.220	0.511	< .001

CI = confidence interval, IVW = inverse variance weighted, MR = Mendelian randomization, OR = odds ratio, SE = standard error, WM = weighted median.

**Table 3:** Reliability test of MR analysis results.

Outcomes	nSNPs	*R* ^2^	*F*	*Q*_*P* value	MR-PRESSO *P* value	Mr pleiotropy *P* value	Egger-intercept
Femoral neck BMD	205	0.099	172.845	1.95e−54	2.16e−29	.923	−2.35e−04
Forearm BMD	215	0.101	81.045	4.23e−10	1.21e−16	.137	−0.005
Lumbar spine BMD	205	0.098	136.568	6.21e−62	1.74e−24	.644	−0.001
Total body BMD	241	0.112	247.833	1.10e−232	7.75e−39	.406	−0.002
Total body BMD (0–15)	236	0.111	109.032	1.96e−22	3.25e−21	.556	−0.002
Total body BMD (15–30)	233	0.110	90.221	7.63e−05	2.76e−18	.747	0.001
Total body BMD (30–45)	238	0.111	150.051	6.12e−28	4.72e−27	.238	−0.004
Total body BMD (45–60)	240	0.112	209.787	5.26e−68	1.50e−34	.544	−0.002
Total body BMD (over 60)	241	0.112	161.168	1.39e−66	1.36e−28	.395	−0.003
Heel BMD	265	0.120	15,240.213	5.03e−08	6.54e−211	0.01	−2.36e−04
Osteoporosis	236	0.110	236.574	1.29e−27	2.10e−37	.342	0.003
Osteoporosis Fracture	236	0.110	90.698	4.74e−10	2.16e−18	.744	0.002

BMD = bone mineral density, MR-PRESSO = Mendelian randomization-pleiotropy residual sum and outlier, *Q*_*P* value = *P* value of Cochran *Q*, nSNPs = number of single nucleotide polymorphisms.

**Figure 6.**
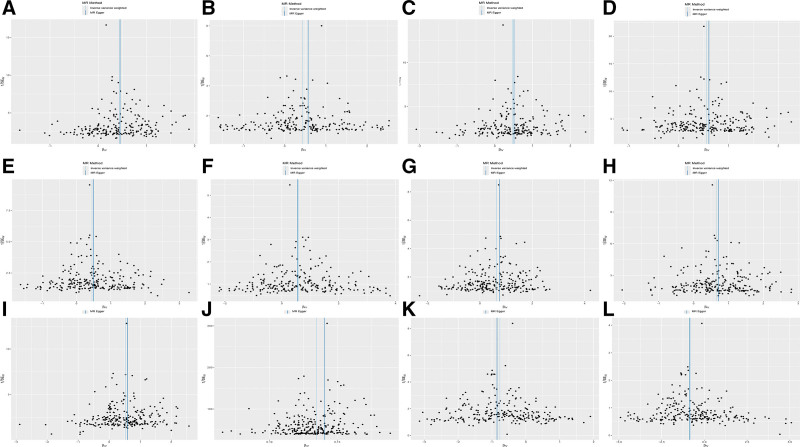
Funnel plots. Funnel plot for the effect of heel broadband ultrasound attenuation, direct entry on (A) femoral neck BMD, (B) forearm BMD, (C) lumbar spine BMD, (D) total body BMD, (E) total body BMD (0–15), (F) total body BMD (15–30), (G) total body BMD (30–45), (H) total body BMD (45–60), (I) total body BMD (over 60), (J) heel BMD, (K) osteoporosis, (L) osteoporosis fracture. BMD = bone density, MR = Mendelian randomization.

Considering the correlates of osteoporosis and osteoporotic fractures, IVW results showed a positive causal relationship between heel broadband ultrasound attenuation and femoral neck BMD (nSNPs = 205, *β* = 0.447, SE = 0.034, OR = 1.154, 95% CI: 1.464–1.670, *P* < .001, *F* = 172.845, MR pleiotropy *P* value = .923) and forearm BMD (nSNPs = 215, β = 0.440, SE = 0.049, OR = 1.552, 95% CI: 1.410–1.708, *P* < .001, *F* = 81.045, MR pleiotropy *P* value = .137) and lumbar spine BMD (nSNPs = 205, *β* = 0.474, SE = 0.041, OR = 1.606, 95% CI: 1.483–1.738, *P* < .001, *F* = 136.568, MR_pleiotropy *P* value = .644). Total body BMD (0–15) (nSNPs = 236, *β* = 0.452, SE = 0.043, OR = 1.572, 95% CI: 1.444–1.711, *P* < .001, *F* = 109.032, MR pleiotropy *P* value = .556), total body BMD (15–30) (nSNPs = 233, *β* = 0.587, SE = 0.062, OR = 1.799, 95% CI: 1.594–2.031, *P* < .001, *F* = 90.221, MR pleiotropy *P* value = .747), total body BMD (30–45) (nSNPs = 238, *β* = 0.634, SE = 0.05, OR = 1.885, 95% CI: 1.703–2.087, *P* < .001, *F* = 150.051, MR pleiotropy *P* value = .238), total body BMD (45–60) (nSNPs = 240, *β* = 0.667, SE = 0.046, OR = 1.949, 95% CI: 1.780–2.133, *P* < .001, *F* = 193.198, MR-pleiotropy *P* value = .544), total body BMD (over 60) (nSNPs = 241 over 60), total body BMD (over 60) (nSNPs = 241, *β* = 0.527, SE = 0.042, OR = 1.694, 95% CI: 1.561–1.837, *P* < .0001, *F* = 161.168, MR-pleiotropy *P* value = −.395), heel BMD (nSNPs = 265, *β* = 0.135, SE = 0.001, OR = 1.144, 95% CI: 1.142–1.147, *P* < .001, *F* = 15,240.213, MR-pleiotropy *P* value < .01). Osteoporosis (nSNPs = 236, *β* = −0.766, SE = 0.050, OR = 0.465, 95% CI: 0.421–0.512, *P* < .001, *F* = 236.574, MR pleiotropy *P* value = .342) and osteoporosis fracture (nSNPs = 236, β = −0.825, SE = 0.087, OR = 0.438, 95% CI: 0.370–0.519, *P* < .001, *F* = 90.698, MR-pleiotropy *P* value = 0.744) there was a negative correlation between (Tables 2 and 3). There was no horizontal pleiotropy in this study (Table 3). Sensitivity analysis showed robust results (Figs. 4, 5 and 6).

**Figure 7.**
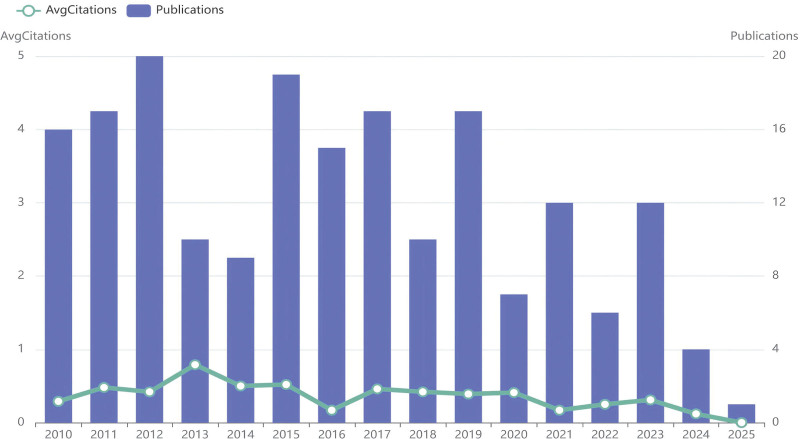
Annual publication volume and average annual citation count. It is usually presented through bar charts or line graphs, with each point representing a year and the height indicating the number of published papers in that year.

The publication volume showed fluctuating growth from 2010 to 2012, declined from 2013 to 2014, and experienced rapid growth from 2015 to 2017. It decreased in 2018 and exhibited fluctuating changes from 2019 to 2025. The total citation count demonstrated fluctuating variations from 2010 to 2025 (Fig.7).

**Figure 8.**
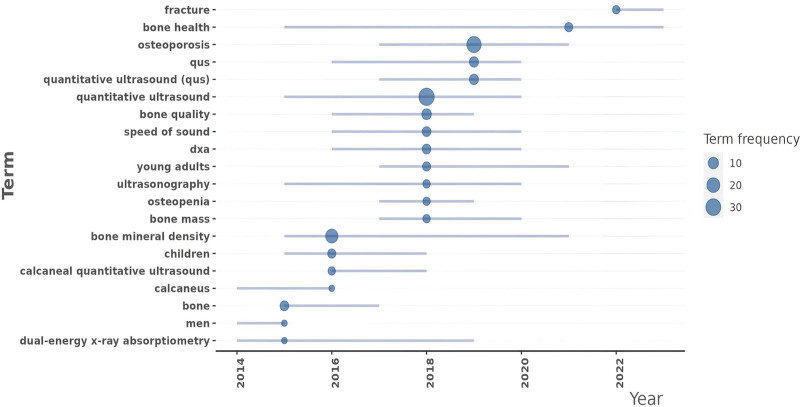
Trending topics. Based on the changes of each topic over different time periods, identify the themes that have undergone the most significant changes or are of the highest concern. DXA = dual-energy X-ray absorptiometry, QUS = quantitative ultrasound.

Trend theme keyword analysis (Fig.8) reveals that as early as 2014 to 2016, the main keywords were represented by DXA, men, and calcaneus. This indicates researchers began investigating the role of dual-energy X-ray in male bone calcium metabolism. From 2016 to 2020, the predominant keywords were BMD, QUS, and osteoporosis. Undoubtedly, researchers had started actively exploring the direction of using QUS to measure bone density for osteoporosis diagnosis. Between 2020 and 2025, the research focus shifted toward bone health and fracture, demonstrating that the study objectives had evolved from simple osteoporosis diagnosis to investigating bone health aspects such as predicting osteoporotic fractures.

**Figure 9.**
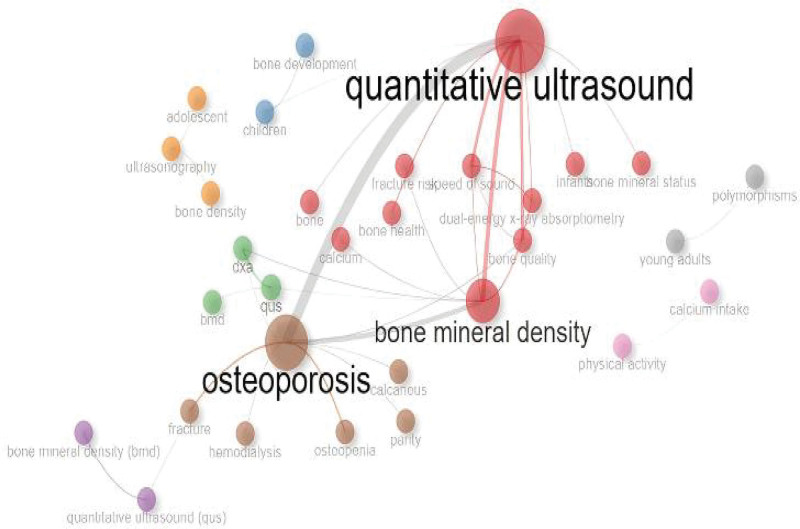
Topic co-occurrence network. A network constructed based on the co-occurrence relationships of keywords or themes in the literature dataset. Identifying the co-occurrence relationships between keywords or themes in the literature.

In Figure 9, we present the co-occurrence network of the most frequent keywords from 192 articles. They are categorized into 3 groups: “QUS,” “BMD,” and “osteoporosis.” Keywords within the same cluster are marked with identical colors and grouped together as they frequently appear in the same articles. The purple cluster contains 2 primary keywords: QUS and BMD. “QUS” is commonly used to measure “BMD.” The red cluster aggregates the largest number of articles, representing fundamental issues related to diagnosing or predicting diseases after QUS detects BMD, such as bone health status and osteoporotic fractures. The green cluster pertains to the diagnosis of bone density, including methods like DXA and QUS. The brown cluster includes common indicators and complications of osteoporosis, such as calcaneus, hemodialysis, osteopenia, and fracture. The blue cluster encompasses changes in bone mass in children, such as bone development. The orange cluster primarily reflects juvenile bone measurement methods, like ultrasonography. The gray cluster is associated with different types of bone density in young adults. The pink cluster relates to interventions for osteoporosis, such as exercise and calcium intake.

## 4. Discussion

Osteoporosis is a common metabolic bone disease characterized by a decrease in bone mass and a decline in bone quality, leading to fragile and fracture-prone bones. The disease is becoming increasingly prevalent globally and is especially prominent in the elderly population. Osteoporosis not only affects patients’ quality of life, but also significantly increases the risk of fracture, which in turn results in high healthcare costs and socioeconomic burden.^[10]^ Current screening and diagnostic tools rely on DXA to measure BMD; however, the limitation of this method is that it does not allow for a comprehensive and in-depth assessment of bone strength as well as potential fracture risk.^[11]^ Therefore, it is particularly important to explore new screening methods for osteoporosis in order to support more effective prevention and treatment. The aim of this study was to investigate the validity of QUS of the heel bone in the prediction of osteoporosis and its associated fractures, especially whether it is independent of BMD. By using MR analysis, we were able to control potential confounders more effectively to provide more reliable causal inferences.^[12,13]^ The results of the study showed that there was a significant correlation between heel QUS and BMD, and that the validity of QUS in predicting osteoporosis and fracture risk was significantly influenced by BMD. This finding provides a new reference point for clinical practice and emphasizes the importance of integrating BMD in osteoporosis risk assessment.^[5,14]^

The key findings of this study showed a significant correlation (*P* < .001) between QUS metrics of the heel bone and BMD, suggesting a degree of overlap between the 2 in the prediction of osteoporosis. This result suggests that heel QUS cannot be used independently of BMD to assess osteoporosis risk, and therefore BMD should be considered as a factor in clinical application. Previous studies have also shown that the accuracy of QUS in assessing osteoporosis patients is directly related to BMD, which is consistent with the findings of our study.^[3,14]^ Although a correlation was observed, further research is needed to establish a combined model of QUS and BMD to further verify the correlation between regions. In addition, the mechanism of interaction between QUS and BMD may involve changes in bone microarchitecture, and further studies are needed to explore how to combine QUS and BMD to improve the prediction of osteoporosis, especially in clinical practice.

In terms of fracture risk prediction, the findings showed that heel QUS was effective in predicting patients with osteoporotic fractures, but its predictive ability was significantly affected by BMD. This finding emphasizes that the value of QUS in fracture risk assessment needs to be combined with BMD results to provide more accurate clinical decision support. It has been shown that the predictive ability of QUS may vary in different populations, suggesting that it needs to be optimized for specific populations in practical applications.^[15,16]^ Future studies could explore how to integrate QUS and BMD into a more accurate fracture risk assessment model to better serve the clinical management of patients.

The application of MR methods enhanced our inference of causality and avoided possible bias in traditional observational studies. By using genetic variants as IVs, we were able to analyze the causal relationship between heel QUS and BMD, osteoporosis, and fracture risk more effectively. This design makes the results more scientific and is an important guide for clinical applications.^[17,18]^ In addition, the application of MR in other fields has shown its broad prospects, and how to optimize this design in future studies will be an important direction.

With the deepening understanding of osteoporosis and its related fractures, the integration of genetics and QUS technology has provided new possibilities for constructing individualized osteoporosis management strategies. Recent studies have shown that genetic factors play a significant role in BMD and the development of osteoporosis. For instance, certain genetic variants are closely associated with changes in BMD, offering a scientific basis for targeted interventions tailored to specific genetic backgrounds.^[19]^ The noninvasive assessment of bone density and quality using QUS technology, when combined with genetic information, can offer more precise guidance for individualized treatment plans.

The construction of individualized osteoporosis management strategies requires consideration of multiple aspects, including genetics, environmental factors, and lifestyle. By analyzing a patient’s genetic background, individuals at higher risk of osteoporosis can be identified, enabling early intervention. For example, studies have shown a significant correlation between low body mass index and low BMD, with these factors being more pronounced in populations with higher genetic susceptibility.^[20]^ Therefore, combining QUS-based BMD assessment with genetic information can assist physicians in developing more personalized prevention and treatment plans, thereby improving the effectiveness of bone health management.

Finally, the findings suggest that relying on QUS alone for osteoporosis screening may lead to underdiagnosis, so it is recommended to use QUS in combination with BMD. This strategy not only improves the identification of early osteoporosis, but also provides better preventive and therapeutic measures for patients. In clinical practice, physicians should take care to combine patients’ BMD data when using QUS to improve the accuracy of screening.^[2,5]^ In the future, the direction of technological development for osteoporosis screening should also focus on how to efficiently integrate different diagnostic tools to achieve comprehensive bone health management. The limitations of this study are mainly characterized by the relatively small sample size and the lack of representation of different ages and genders. In addition, the lack of clinical validation analysis may have affected the broad applicability of the results. The dataset may have interlot variations, which may lead to biased results, thus affecting the accurate assessment of the relationship between QUS of the heel bone and BMD. Therefore, future studies should consider conducting them in larger, diverse samples to enhance understanding and application in this area.

In summary, this study reveals that QUS of the heel bone is not independent of the effect of BMD in predicting osteoporosis and fracture risk, emphasizing the importance of integrating BMD in clinical assessment. This finding provides a new perspective on the screening and management of osteoporosis and suggests that the complex relationship between the 2 should be further explored in the future in order to optimize clinical decision-making and improve patient prognosis.

## 5. Conclusion

In this study, we conducted a bibliometric review of MR research in the fields of QUS diagnosis of BMD, osteoporosis, and osteoporotic fractures. Through systematic analysis of relevant literature, we explored key research trends in this domain, including genetic factors, environmental influences, and the role of MR in QUS diagnosis of BMD, osteoporosis, and osteoporotic fractures. The findings demonstrate that MR serves as an effective tool for evaluating causal relationships in QUS diagnosis of BMD, osteoporosis, and osteoporotic fractures, providing crucial insights for personalized treatment strategies. Our results indicate that interdisciplinary holistic research approaches combining MR with other methodologies such as bioinformatics are essential for advancing QUS diagnosis of osteoporosis research and developing personalized treatment plans.

## Acknowledgements

We acknowledge the investigators and participants of the original GWAS. We are grateful for the GWAS sharing of the summary data used in this study.

## Author contributions

**Data curation:** Yihua DuMei.

**Writing – original draft:** Zhu Na, Xinyu Guo, Yihua DuMei.

**Writing – review & editing:** Zhu Na, Xinyu Guo, Yihua DuMei.
























